# Exploring the Application of Advanced Chromatographic Methods to Characterize the Surface Physicochemical Properties and Transition Phenomena of Polystyrene-*b*-poly(4-vinylpyridine)

**DOI:** 10.3390/molecules29204812

**Published:** 2024-10-11

**Authors:** Tayssir Hamieh

**Affiliations:** 1 Faculty of Science and Engineering, Maastricht University, P.O. Box 616, 6200 MD Maastricht, The Netherlands; t.hamieh@maastrichtuniversity.nl; Tel.: +31-6-5723-9324; 2 Laboratory of Materials, Catalysis, Environment and Analytical Methods (MCEMA), Faculty of Sciences, Lebanese University, Beirut P.O. Box 6573/14, Lebanon; 3 Institut de Science des Matériaux de Mulhouse, Université de Haute-Alsace, CNRS, IS2M UMR 7361, F-68100 Mulhouse, France

**Keywords:** adsorption, London dispersion interaction, dispersive and polar free surface energy, enthalpy and entropy of adsorption, Hamieh thermal model, glass transition, Lewis’s acid–base surface energies

## Abstract

The linear diblock copolymer polystyrene-*b*-poly(4-vinylpyridine) (PS-P4VP) is an important copolymer recently used in many applications such as optoelectronics, sensors, catalysis, membranes, energy conversion, energy storage devices, photolithography, and biomedical applications. (1) Background: The surface thermodynamic properties of PS-P4VP copolymers are of great importance in many chemical and industrial processes. (2) Methods: The inverse gas chromatography (IGC) at infinite dilution was used for the experimental determination of the retention volumes of organic solvents adsorbed on copolymer surfaces as a function of temperature. This led to the variations in the free energy of interaction necessary to the evaluation of the London dispersive and polar acid–base surface energies, the polar enthalpy and entropy, the Lewis acid–base constants, and the transition temperatures of the PS-P4VP copolymer. (3) Results: The application of the thermal Hamieh model led to an accurate determination of the London dispersive surface energy of the copolymer that showed non-linear variations versus the temperature, highlighting the presence of two transition temperatures. It was observed that the Lewis acid–base parameters of the copolymer strongly depend on the temperature, and the Lewis base constant of the solid surface was shown to be higher than its acid constant. (4) Conclusions: An important effect of the temperature on the surface thermodynamic properties of PS-P4VP was proven and new surface correlations were determined.

## 1. Introduction

Block copolymers are considered as a special class of polymers in the large family of soft matter [1], consisting of at least two fragments of different chemical nature of the polymer, joined together by a junction—type of covalent bond—and which can be easily synthetized by various polymerization techniques [2,3,4,5]. The advantage of the block copolymers resides in the coupling of two polymers and combining their divergent properties differing in a single structure. Block copolymers are widely used as self-assembling polymer materials that provide access to a variety of periodic nanoscale morphologies with feature sizes ranging from 5 to 50 nm [6,7].

Many works were devoted to block copolymers because of their self-assembly into 2- or 3-dimensional periodic nanostructures, such as spherical, cylindrical, lamellar, and gyroid structures [8,9,10,11,12], and the crucial interest of the control of their surface structure and uses in many areas of science and technology where surface properties play an important role due to their uniformity, spatial regularity at the nanometric scale [13], and versatile nanoscale fabrication tool for semiconductor devices and other applications [14,15]. The block copolymers have received considerable attention due to their ability to microphase-separate into various nanostructures when studied in bulk and/or thin films. The important contribution to their self-assembly results in the immiscibility between the different covalently connected segments and their application towards the fabrication of high-resolution patterns for nanolithography applications. Recent advances in directed self-assembly of block copolymers enable low defect density, extremely minimal dimensions, facile processability, etching selectivity, a low cost, and the ability to design various patterns [16,17]. The periodic ordered microphase separation structures at the molecular chain scale formed by block copolymers are ideal materials for self-assembly of structured particles and for fabricating polymer particles with adjustable shapes, internal phase structures, and surface characteristics [18,19].

The block copolymers, due to their high periodicity, features, and precise size, are used in many applications such as optoelectronics, photonics, sensors, field emission, catalysis, membranes, energy conversion, energy storage devices, photolithography, nanomedicine, and biomedical applications for the diagnosis and treatment of a variety of diseases [20,21,22,23,24,25,26,27,28,29,30,31,32,33,34]. They also serve as templates for the deposition of inorganic materials [25]. The solvent-mediated infiltration of different-oxidation-state metal cations selectively inserted into self-assembled block copolymer nanopatterns formed from polystyrene-*b*-poly(4-vinylpyridine) (PS-*b*-P4VP) thin films. It was observed that the stability of the as-obtained metal–pyridine complex is highly influenced by the coordination chemistry of the metal ion and the P4VP group and this impacts the formation of metal oxide patterns [26]. Shevate et al. [35] used the PS-*b*-P4VP copolymer to obtain isoporous block copolymer membranes by non-solvent induced phase separation and showed a highly ordered surface layer, high flux, and superior separation properties and a strong flux dependence of pH; pores closed at low pH and opened at high pH. A unique perforated lamellar (PL) morphology was observed by Singh et al. [36] in a mixture of an asymmetric PS-*b*-P4VP block copolymer and CdSe–CdS quantum dots (QDs). The PL morphology formed by the PS-*b*-P4VP/CdSe–CdS composites consisted of alternating layers of PS and P4VP, where the layer formed by the minority PS block contained cylindrical perforations of the majority P4VP block. The thermal and rheological properties of PS-*b*-P4VP diblock copolymers were studied by Xue et al. [37] and Schultze et al. [38] in order to obtain information about the optimum foaming temperature. PS*-b-*P4VP was also used to identify the constructional details of the polymer thin film morphology [39].

The important and interesting uses and applications of PS*-b-*P4VP copolymers, particularly in the field of nanotechnology, nanolithography, nanodevices, and materials science, imply the necessity of the determination of the surface physicochemical properties of PS*-b-*P4VP copolymers. Because of the important lack in this domain, this research work was devoted to an accurate evaluation of the surface thermodynamic properties of this copolymer, such as the London dispersive surface energy, the free dispersive and polar interaction energy, the Lewis acid–base parameters, and the transition temperatures of PS*-b-*P4VP diblock copolymers as a function of the temperature. To carry out that, the inverse gas chromatography (IGC) technique at infinite dilution [40,41,42,43,44,45,46,47,48,49,50,51,52] was used by applying our new methodology based on the Hamieh thermal model and the London dispersion interaction that were proven to give more accurate surface parameters of solid surfaces [53,54,55,56,57,58,59,60,61,62,63]. This methodology corrected the values of the London dispersive energy of solid surfaces previously obtained by several authors using classic methods [64,65,66,67,68,69,70,71,72,73,74,75,76,77,78,79,80,81,82,83,84].

## 2. Results

### 2.1. Variations in RTlnVn(T) of Solvents Adsorbed on PS-b-P4VP Diblock Copolymer

**Figure 1:**
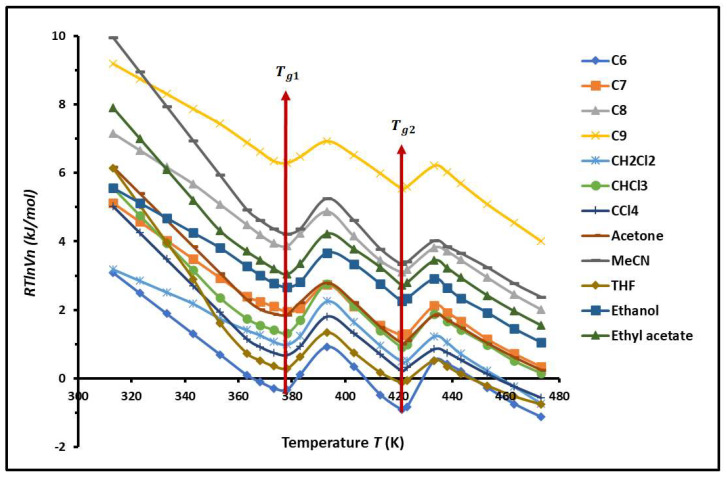
Variations in RTlnVn (kJ/mol) of n-alkanes and polar solvents adsorbed on the PS-*b-*P4VP diblock copolymer as a function of temperature.

The values of RTlnVn(T) of n-alkanes and polar solvents adsorbed on the PS-*b*-P4VP diblock copolymer are given in Tables S1 and S2 (Supplementary Materials) and the variations are plotted in Figure 1.

The non-linear evolution of RTlnVn of the different organic molecules adsorbed on the PS-P4VP diblock copolymer against the temperature, drawn in Figure 1, showed the presence of two minima for all used solvents at the respective temperatures $T_{g1}=378.15K$ and $T_{g2}=421.15K$. These two particular temperatures perfectly correspond to the respective transition temperatures of PS and P4VP. This interesting result proved that the block copolymers behave as separated phases.

The transition temperatures of the PS-*b*-P4VP diblock copolymer were studied by several authors using differential scanning calorimetry (DSC) [38,85,86,87,88,89,90]. Zhang et al. [85] showed that the pure PSb-P4VP copolymer exhibits two glass transition temperatures: one $T_{g}$ of PS is 100.88 °C and the other is the $T_{g}$ of P4VP at 130.98 °C, largely depending on the ratio of the molecular masses of PS on P4VP blocks. However, other values of $T_{g}$ of P4VP ($T_{g}$ = 138 °C) were shown by Zhao et al. [86]. The ratio of number molecular weights of PS on P4VP varied between 30% and 50%. 

However, the glass transition temperature $T_{g}$ of the PS and P4VP of the diblock copolymer was analyzed by Schulze et al. [38] using the DSC technique. They highlighted two glass transitions of approximately 105 °C for polystyrene and 150 °C for poly(4-vinylpyridine). The same values of $T_{g}$ were obtained by Rahikkala et al. [74]. The DSC measurements on the PSb-P4VP block copolymers carried out by Huang et al. [90] also exhibited two glass transitions, indicating that the resulting block copolymers are phase-separated and reporting two $T_{gs}$ for PS and P4VP blocks in the PSb-P4VP copolymer respectively equal to 100 °C, and 150 °C for a ratio of number molecular weights of PS on P4VP equal to 20%. These two transition temperatures are obviously quite similar to those of respective homopolymers, indicating that the resulting block copolymers are phase-separated in the condensed state [90].

The results of $T_{g}$ obtained by this work $(T_{g1}=105\;{}^{\circ}C$ and $T_{g2}=148\;{}^{\circ}C)$ are very close to those obtained by Schulze et al. [38], Rahikkala et al. [89], and Huang et al. [90] with a small deviation not exceeding 5% due to the difference observed in the molecular weight and composition of the blocks.

### 2.2. London Dispersive Surface Energy of PS-b-P4VP Diblock Copolymer

**Figure 2:**
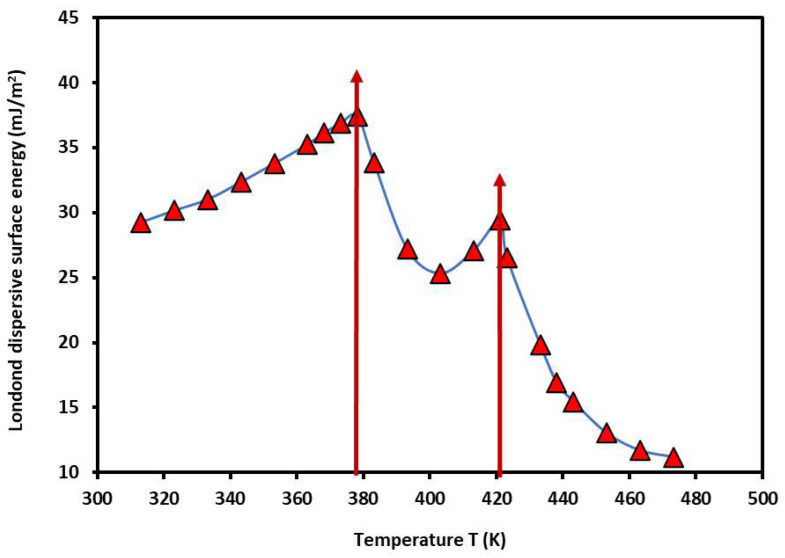
Variations in the London dispersive surface energy $\gamma_{s}^{d}\;(mJ/m^{2})$ of the PS*-b-*P4VP diblock copolymer as a function of the temperature *T* (K) using the Hamieh thermal model. The vertical red arrows indicate the transition temperatures of the copolymer.

Using the results given in Tables S1 and S2, and the Hamieh thermal model [53,54,55,56], it was possible to determine the London dispersive surface energy $\gamma_{s}^{d}(T)$ of the PS-*b*-P4VP diblock copolymer as a function of the temperature. The results are given in Figure 2. 

The non-linear variations in the London dispersive surface energy showed two maxima corresponding to the two glass transition temperatures of PS and P4VP blocks in the PS-*b*-P4VP diblock copolymer, then confirming those previously obtained in Figure 1:

- $T_{g1}=105\;{}^{\circ}C$ relative to PS glass transition.
- $T_{g2}=148\;{}^{\circ}C$ relative to P4VP glass transition.

In several previous studies [50,61,62,63], the transition temperatures of some polymers in bulk and adsorbed phases were highlighted by the IGC technique. An important effect of the temperature, the polymer tacticity, and the recovery fraction was shown on the values of the second-order transition temperatures. The variations in $\gamma_{s}^{d}(T)$ of the PS-*b*-P4VP diblock copolymer are constituted by three parabolic functions given in Table 1 and represented by the following general equation with an excellent linear regression coefficient (R^2^ > 0.99):

(1) $$\gamma_{s}^{d}\left(T\right)=aT^{2}+bT+c$$

The results in Table 1 confirmed the general tendency of the variations in the London dispersive surface energy of polymers obtained in the case of transition phenomena of Poly(methyl methacrylate) (PMMA) [50,61,62,63]. For the PS-*b*-P4VP diblock copolymer, it was proven that the two glass transition temperatures correspond to those of PS and P4VP separately taken with slight variation. In the case of first-order linear interpolation in the considered temperature interval [313.15 K–473.15 K], a bad linear correlation, R^2^ = 0.6456, was obtained, certainly due to non-linearity of the function $\gamma_{s}^{d}(T)$. The corresponding $\gamma_{s}^{d}(T)$ of the PS*-b-*P4VP diblock copolymer is given by the following relation:

(2) $$\gamma_{s}^{d}\left(T\right)=-0.147T+84.50$$

The useful information obtained by this large approximation was the determination of the following surface energetic parameters of the PS*-b-*P4VP diblock copolymer:

- The surface entropy: $S_{s}=-\frac{{d\gamma }_{s}^{d}}{dT}=0.147\;mJ\times m^{-2}\times K^{-1}$;
- The London dispersive energy at 0K: $\gamma_{s}^{d}\left(0K\right)=84.50\;mJ\times m^{-2}$;
- The intrinsic temperature $T_{int.}=\gamma_{s}^{d}\left(0K\right)/S_{s}=574.8\;K$.

The above values can be compared to those obtained in other works on PMMA [61,62,63], with smaller surface parameter values of the PS-*b*-P4VP diblock copolymer, but higher intrinsic temperature.

### 2.3. Polar Free Surface Energy of PS-b-P4VP Diblock Copolymer

**Figure 3:**
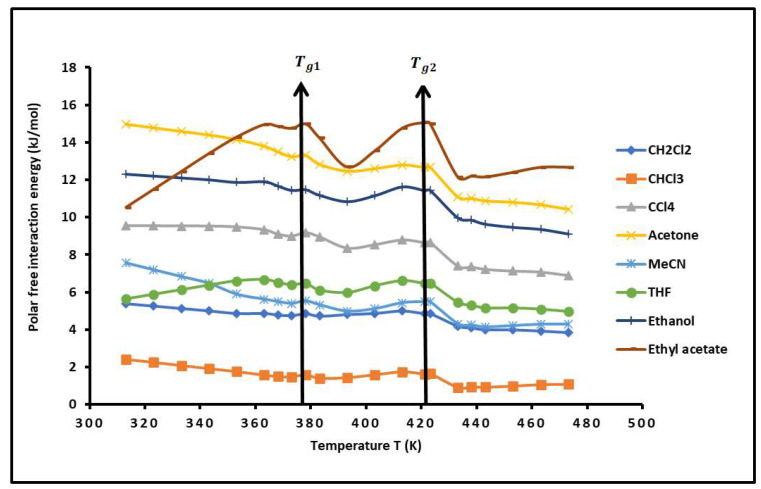
Evolution of polar free interaction energy $\left(-∆G_{a}^{p}\left(T\right)\right)$ (kJ/mol) of different polar solvents adsorbed on PS*-b-*P4VP diblock copolymer.

By applying our new methodology, using the London dispersion interaction equation, the results given in Tables S1 and S2, and the values of the deformation polarizability $\alpha_{0X}$ and the ionization energies [91] of the various n-alkanes and polar molecules adsorbed on the PS*-b-*P4VP diblock copolymer led to the variations in the free polar surface energy ($-∆G_{a}^{p}\left(T\right)$) of polar solvents adsorbed on the copolymer as a function of the temperature. The values of $-∆G_{a}^{p}\left(T\right)$ are given in Table S3 and the curves are plotted in Figure 3. A large difference in the values of the free polar energy was observed with the various polar solvents adsorbed on the PS*-b-*P4VP diblock copolymer. The polar probes can be globally classified in increasing order of their polar interaction energy,

CHCl_3_ < CH_2_Cl_2_ < THF < MeCN < CCl_4_ < ethyl acetate < ethanol < acetone

showing the highest polar free energy with Lewis’s base solvents. 

Non-linear variations in $-∆G_{a}^{p}\left(T\right)$ of the different polar molecules were also observed due to the reorganization of the surface groups of the copolymer blocks and highlighted the two glass transition temperatures of the PS-*b*-P4VP diblock copolymer with small fluctuations in the polar interaction energy as a function of the temperature (Figure 3).

### 2.4. Polar Enthalpy and Entropy of Adsorption

**Figure 4:**
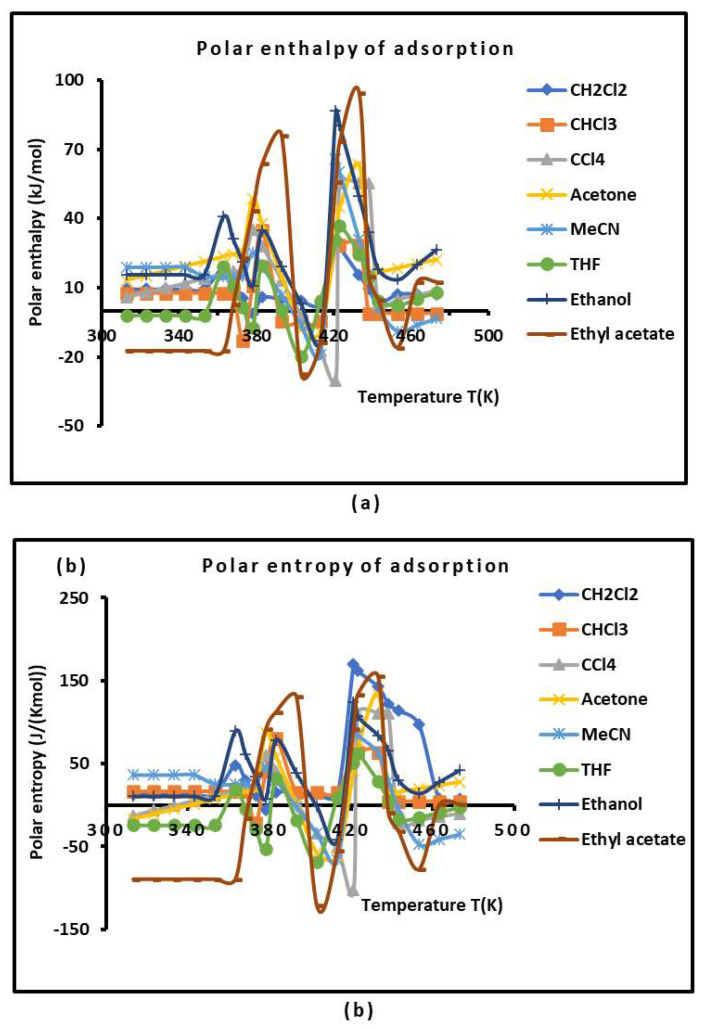
Variations in the polar interaction enthalpy $\left(-∆H_{a}^{p}\left(T\right)\;(kJ/mol\right)$ (**a**) and entropy ($-∆S_{a}^{p}\left(T)\right)\;(JK^{-1}{mol}^{-1})$ (**b**) of polar solvents adsorbed on the PS-*b*-P4VP diblock copolymer.

The results presented in Table S3 giving the non-linear variations in $-∆G_{a}^{p}\left(T\right)$ of adsorbed solvents on the PS*-b-*P4VP diblock copolymer allowed for determining the values of the polar enthalpy ($-∆H_{a}^{p}\left(T\right))$ (Table S4) and entropy ($-∆S_{a}^{p}\left(T)\right)$ (Table S5) of the adsorption of polar probes on the copolymer as a function of the temperature. The results are plotted in Figure 4. The variations in $-∆H_{a}^{p}\left(T\right)$ showed the adsorption and desorption phenomena of the polar molecules on the copolymer surface with a stronger tendency to the adsorption and sever and non-linear fluctuations as a function of the temperature due to the presence of the glass transition affecting the group reorganization and rearrangement when the temperature is very close to the transition phenomena. However, brutal variations in the polar entropy change ($-∆S_{a}^{p}\left(T)\right)$ of the adsorption had a certain disorder near the transition temperatures and a more ordered and organized surface of the copolymer far from these transition temperatures. Some solvents such as THF and ethyl acetate showed higher disorder of interaction with the PS-*b*-P4VP diblock copolymer. The maximum of disorder was observed at the two transition temperatures for all polar molecules. 

The non-linearity variations ($-∆H_{a}^{p}\left(T\right)$ and $-∆S_{a}^{p}\left(T)\right)$ of the different adsorbed solvents observed as a function of temperature led to important variations in the Lewis acid–base properties of the PS*-b-*P4VP diblock copolymer against the temperature. However, the calculations of the average values ($-∆H_{a}^{p}$ and $-∆S_{a}^{p})$ of the various polar molecules allowed for obtaining the results in Table 2.

The results in Table 2 show that the linear regression coefficients are generally very bad because of the extreme non-linearity previously observed in Figure 3 and Figure 4.

### 2.5. The Temperature Effect on the Lewis Acid–Base Parameters of the Copolymer 

**Figure 5:**
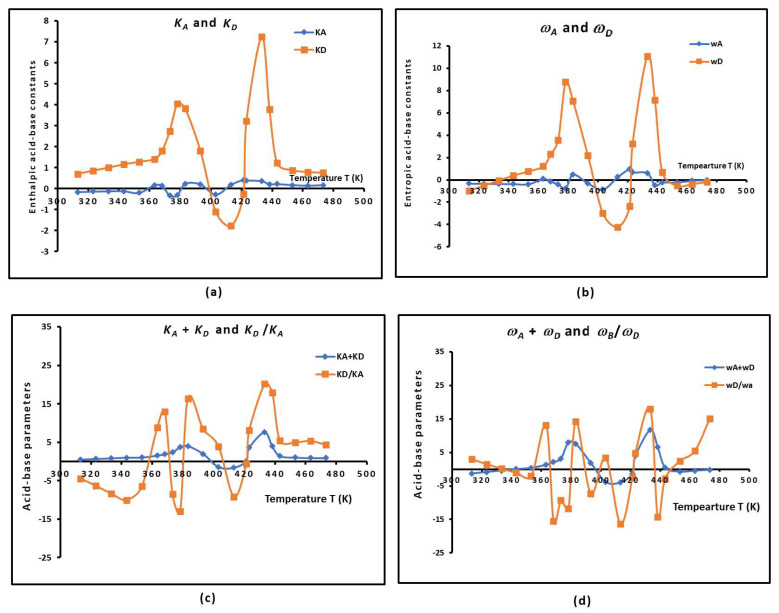
Variations in the various enthalpic and entropic acid–base parameters of the PS*-b-*P4VP diblock copolymer as a function of the temperature—(**a**): $K_{A}$ and $K_{D}$, (**b**): $\omega_{A}$ and $\omega_{D}$, (**c**): $K_{D}$*/*$K_{A}$ and $\left(K_{D}+K_{A}\right)$, (**d**): $\omega_{D}\;$*/*${\;\omega }_{A}$ and ${(\omega }_{D}+\omega_{A})$. The values of $\omega_{A}$ and $\omega_{D}$ given in the figures were multiplied by 10^3^.

Using the values of $-∆H_{a}^{p}\left(T\right)\;$and $-∆S_{a}^{p}\left(T\right)$ given in Tables S4 and S5, the author determined the Lewis enthalpic acid–base parameters $K_{A}$ and $K_{D}\;$and the entropic acid–base parameters $\omega_{A}$ and $\omega_{D}\;$of the PS*-b-*P4VP diblock copolymer as a function of the temperature. The evolution of the various enthalpic and entropic acid–base parameters of the PS-*b*-P4VP diblock copolymer as a function of the temperature is plotted in Figure 5. It was shown that the diblock copolymer exhibited very stronger Lewis base character, reaching at certain temperatures 20 times higher than its Lewis acidity. The basicity of the copolymer was proven to be maximum at the glass transition. An important non-linearity of the different Lewis acid–base parameters regarding the PS-*b*-P4VP diblock copolymer was observed (Figure 5) when the temperature varied. This is essentially due to the presence of the glass transitions that participate in the acid and base group reorganization, controlling at the same time the variations in the acid–base properties of the copolymer.

The calculations of the average values of $-∆H_{a}^{p}\left(T\right)\;$and $-∆S_{a}^{p}\left(T\right)$ of the adsorbed polar molecules gave the following average values of the acid–base constants independently from the temperature (Table 3).

Table 3 shows that the PS-*b*-P4VP diblock copolymer then exhibits a stronger Lewis base character, which is globally 7.5 times more basic than acidic. The evolution of the Lewis acid–base of the copolymer also highlighted the presence of the two glass transition temperatures.

### 2.6. Separation Distance, Lewis Acid–Base Surface Energies of PS-b-P4VP Copolymer, and Polar Surface Energy of Solvents

**Figure 6:**
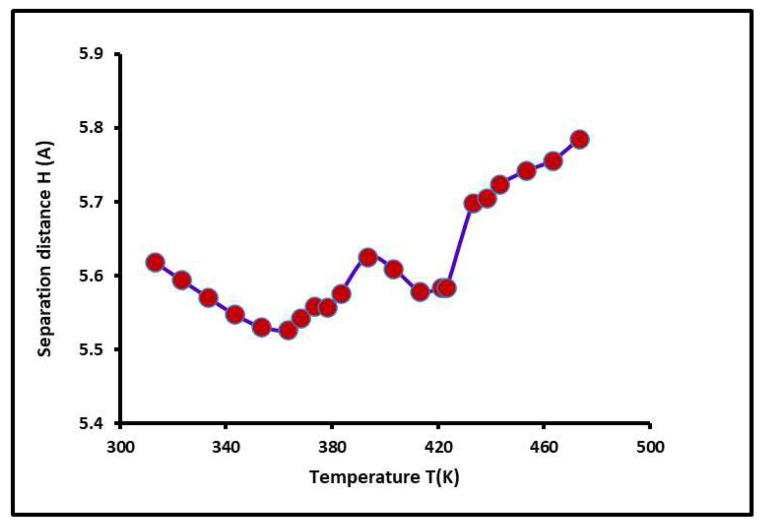
Variations in the separation distance *H* (*T*) (Å) of the PS*-b-*P4VP diblock copolymer as a function of the temperature *T* (K).

The values of the average separation distance H between the solvents adsorbed on the copolymer surface were determined from experimental results. The variations in the separation distance H(T) are plotted in Figure 6 as a function of the temperature. Even if these variations are limited between 5.5 and 6.0 Å, however, it is shown in Figure 6 that the non-linear evolution of H(T) decreased before reaching each glass transition of the PS-*b*-P4VP diblock copolymer and then increased for larger temperatures.

Furthermore, the Lewis acid $\gamma_{s}^{+}$ and base $\gamma_{s}^{-}$ surface energies of the PS-*b*-P4VP diblock copolymer were determined using the Van Oss method, whereas the polar acid–base surface energy $\gamma_{s}^{AB}=\gamma_{s}^{p}$ of the copolymer was obtained from the geometric mean of the polar acid and base energies of the copolymer. Meanwhile, the value of the Lifshitz–van der Waals (LW) surface energy $\gamma_{s}^{LW}=\gamma_{s}^{tot.}$ (or total surface energy of the copolymer) was deduced from the summation of the dispersive and polar energies of the copolymer. This led to determining the variations in the various polar acid and base surface energies, $\gamma_{s}^{+}$, $\gamma_{s}^{-}$, $\gamma_{s}^{p}$, and $\gamma_{s}^{tot.}$, of the copolymer (Table 4).

The comparison between the different surface energy components of the copolymer showed the highest values of polar acid surface energy due to the highest basic character of the surface groups of the copolymer blocks. The results in Table 4 show that the London dispersive surface energy is equivalent to half of the polar surface energy of the PS-*b*-P4VP diblock copolymer, whereas the lowest surface energy was obtained for the basic surface energy component, because of the weaker acid force of the surface groups of the copolymer. 

**Figure 7:**
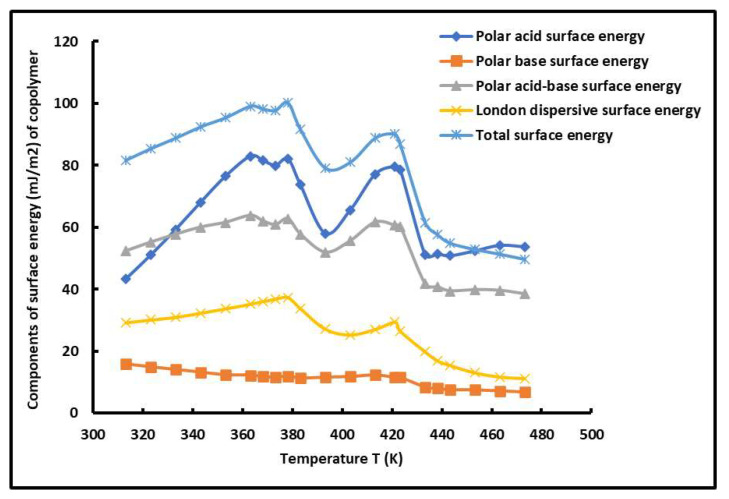
Evolutions of the different components of surface energies $\gamma_{s}^{+}$, $\gamma_{s}^{-}$, $\gamma_{s}^{p}$, $\gamma_{s}^{d}$, and $\gamma_{s}^{tot.}$ (mJ/m^2^) of the PS*-b-*P4VP diblock copolymer as a function of the temperature.

The variations in the various surface energy components are plotted in Figure 7.

The curves of the different components of the surface energy of the copolymer shown in Figure 7 also confirmed the presence of the two glass transition temperatures of the PS-*b*-P4VP diblock copolymer. The same conclusions were observed in several previous studies [61,62,63,92].

On the other hand, the experimental results previously obtained allowed for determining the polar free energy $\left(-∆G_{a}^{p}\left(X\right)\right)$ of a polar molecule denoted by X having a surface area $a_{X}$ and a polar component of the surface energy $\gamma_{l}^{p}$, given by Equation (3):

(3) $$(-∆G_{a}^{p}\left(X\right))=2Na_{X}\sqrt{\gamma_{s}^{p}\gamma_{l}^{p}}$$

Knowing the polar surface energy $\gamma_{s}^{p}$ of the PS-*b*-P4VP diblock copolymer, the polar surface energy of the organic molecules was determined by Equation (4):

(4) $$\gamma_{l}^{p}=\frac{{\left(-∆G_{a}^{p}\left(X\right)\right)}^{2}}{4N^{2}{a_{X}}^{2}\gamma_{s}^{p}}$$

**Figure 8:**
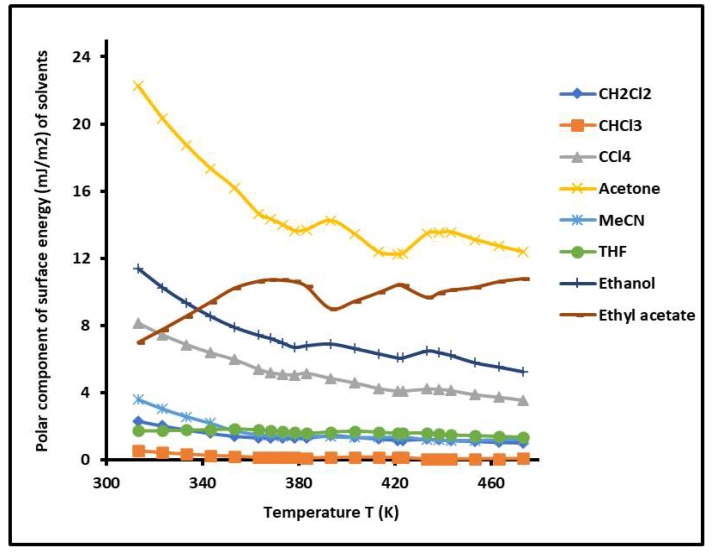
Variations in the polar component of the surface energy $\gamma_{l}^{p}(T)$ (mJ/m^2^) of the different polar solvents adsorbed on the PS-*b*-P4VP diblock copolymer as a function of the temperature.

By varying the temperature, the $\gamma_{l}^{p}$ of the various polar solvents adsorbed on the copolymer was determined versus the temperature. The results are plotted in Figure 8. The highest polar contribution of the surface energy of polar molecules was obtained by acetone, respectively followed by ethyl acetate, ethanol, and carbon tetrachloride. A decrease in the values of the $\gamma_{l}^{p}$ of the various adsorbed polar solvents was observed when the temperature increased with some variations near the glass temperatures of the copolymer. The curves of Figure 8 highlight the highest polar surface energy of acetone, certainly due to the highest polarity of this polar solvent.

### 2.7. Work of Adhesion of Solvents on PS-b-P4VP Copolymer against Temperature

To determine the polar work of adhesion $W_{a}^{p}\left(Copolymer-X\right)$ of the polar organic molecule X adsorbed on the copolymer, the following relation was used:

(5) $$W_{a}^{p}\left(Copolymer-X\right)=2\sqrt{\gamma_{s}^{p}(Copolymer)\gamma_{l}^{p}(X)}$$

**Figure 9:**
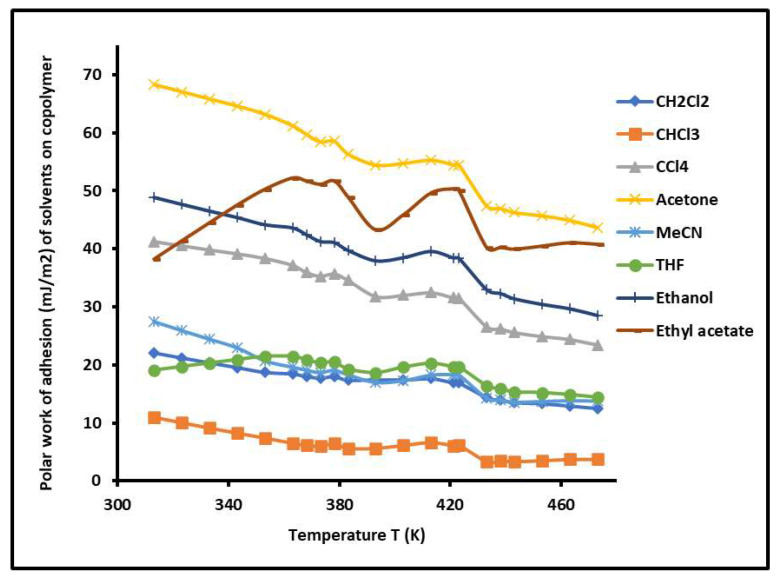
Evolution of $W_{a}^{p}\left(Copolymer-X\right)$ (mJ/m^2^) of polar molecules adsorbed on PS*-b-*P4VP copolymer.

The variations in $W_{a}^{p}\left(Copolymer-X\right)$ versus the temperature are plotted in Figure 9. The plotted curves show non-linear variations in the polar work of adhesion with the presence of two maxima at two particular temperatures corresponding to the two glass temperatures of the PS-*b*-P4VP diblock copolymer.

It was observed that the highest polar work of adhesion corresponded to the adhesion of acetone on the copolymer, followed by ethyl acetate, ethanol, and CCl_4_.

## 3. Materials and Methods

### 3.1. Solvents and Materials

All chemicals were purchased from Sigma-Aldrich (Beirut, Lebanon): the non-polar organic solvents such as *n*-Hexane, *n*-heptane, *n*-octane, and benzene, and the polar molecules, such as dichloromethane, chloroform, and carbon tetrachloride (Lewis’s acid solvents); ethyl acetate, acetone, and tetrahydrofuran (Lewis’s base solvents); and ethanol and acetonitrile (amphoteric solvents). The polystyrene-*b*-poly(4-vinylpyridine) that was previously synthetized [92] through atom transfer radical polymerization (ATRP) with a number average molecular weight equal to Mn = 41,000 g mol^−1^ was from molecular weights of PS and P4VP blocks respectively equal to Mn = 41,000 and 5200 g mol^−1^. The surface energy of n-alkanes and polar solvents were determined as a function of temperature using the Wilhelmy plate tensiometer [50,53,93].

### 3.2. Inverse Gas Chromatography

The net retention time of organic solvents adsorbed on the polystyrene-*b*-poly(4-vinylpyridine) copolymer was determined at different temperatures using inverse gas chromatography at infinite dilution with the help of a Focus GC gas chromatograph equipped with a flame ionization detector of high sensitivity (Sigma-Aldrich, Paris, France). A mass of 1 g of the PS*-b-*P4VP diblock copolymer was packed into a stainless-steel column of a length of 30 cm and 2 mm internal diameter. Helium was used as carrier gas with a flow rate equal to 25 mL/min. The retention times of the different injected organic solvents, measured at infinite dilution, led to the interactions between the organic molecules and the polymer, supposing that there is no interaction between the probe molecules themselves. The column temperatures varied from 30 to 200 °C. Average retention times and volumes were determined by repeating each solvent injection three times with a standard deviation less than 1% in all chromatographic measurements.

### 3.3. Thermodynamic Methods

#### 3.3.1. Fundamental Equation of IGC

The fundamental equation of the inverse gas chromatography at infinite dilution can be written as follows:

(6) $$∆G_{a}^{0}\left(T\right)=-RTlnVn(T)+C(T)$$

where $∆G_{a}^{0}(T)$ is the free energy of the adsorption of organic solvents on the solid materials, Vn is the net retention volume, T is the absolute temperature, R is the perfect constant gas, and C(T) is a constant depending on the temperature and the interaction solvents–sorbent.

The IGC experiments allow for obtaining the values of RTlnVn(T) of the adsorbed probes at different temperatures and consequently the free energy of interaction.

When non-polar solvents such as n-alkanes are adsorbed on the copolymer, $∆G_{a}^{0}(T)$ is only equal to the London dispersive energy $∆G_{a}^{d}(T)$ at any temperature, and one writes

(7) $$∆G_{a}^{0}\left(T\right)=∆G_{a}^{d}(T)$$

However, by using polar solvents, Equation (3) can therefore be written:

(8) $$∆G_{a}^{0}\left(T\right)=∆G_{a}^{d}(T)+∆G_{a}^{p}(T)$$

with $∆G_{a}^{p}(T)$ being the free polar energy of the polar probes.

#### 3.3.2. London Dispersive Surface Energy of Solid Surfaces

In a first attempt, Dorris-Gray [94] determined the London dispersive component $\gamma_{s}^{d}$ of the surface energy of the solid material using Fowkes’s relation [95]. The obtained relation giving $\gamma_{s}^{d}(T)$ is the following:

(9) $$\gamma_{s}^{d}=\frac{{\left[RTln\left[\frac{V_{n}\left(C_{n+1}H_{2(n+2)}\right)}{V_{n}\left(C_{n}H_{2(n+1)}\right)}\right]\right]}^{2}}{4N^{2}a_{-CH2-}^{2}\gamma_{-CH2-}}$$

where $C_{n}H_{2(n+1)}$ and $C_{n}H_{2(n+1)}$ are two consecutive n-alkanes, $a_{-CH2-}$; the surface area of the methylene group with $a_{-CH2-}=6Å$, independent from the temperature; and the surface energy $\gamma_{-CH2-}$ of the methylene group is given by

(10) $$\gamma_{-CH2-}\left(inmJ/m^{2}\right)=52.603-0.058T\;\left(T\;in\;K\right)$$

A second method using the same Fowkes concept was proposed in study [44] and also led to $\gamma_{s}^{d}$ of solid surfaces (Equation (11)):

(11) $$RTln(Vn)=2Na{\left(\gamma_{l}^{d}\gamma_{s}^{d}\right)}^{1/2}+\alpha (T)$$

where a is the surface area of an adsorbed molecule (previously supposed constant), N is the Avogadro number, and α(T) is a constant only depending on the temperature and the solid substrate.

An initial criticism was addressed to the above methods by Hamieh et al. [50] that proved that the London dispersive surface energy of the solvents $\gamma_{l}^{d}$ depended on the temperature and they proposed several molecular models allowing the determination of the surface areas of molecules and then the surface area of the methylene group for each model. The following models were used: the geometric model based on the real form of molecules, cylindrical model based on the cylindrical form of molecules, and spherical model based on the spherical form of molecules. Kiselev results, the two-dimensional van der Waals (VDW) equation, and the two-dimensional Redlich–Kwong (R-K) equation were also included [50].

The second serious criticism was formulated by the Hamieh thermal model, proving in recent works that the surface area of organic solvents strongly depends on the temperature [53,54,55,56,59,60], and this thermal influence effectively modifies the different values of the surface parameters of solid substrates such as $\gamma_{s}^{d}$, $∆G_{a}^{p}$, and the Lewis acid–base surface energies and variables. New equations giving the variation in the surface area $a\left(T\right)$ of polar and non-polar molecules were proposed against the temperature, as well as the expression of surface area of methylene group $a_{-CH2-}\left(T\right)$ [53,54,55,56,59,60]. The linear relations of $\gamma_{l}^{d}$ of the varieties were also given.

The surface energetic parameters of the PS*-b-*P4VP diblock copolymer were determined using our new thermal model, taking into account the variations in $a\left(T\right)$ and $\gamma_{l}^{d}(T)$ of the probes versus the temperature. 

#### 3.3.3. London Dispersive and Polar Free Energies of Adsorption

Several chromatographic methods were used in the literature to determine the London dispersive and polar free energies of solids [41,44,45,46,47,48,49,50]. The various methods were based on the use of several reference thermodynamic parameters such as the boiling point $T_{B.P.}$ of the solvents [41], and the vapor pressure $P_{0}$ of the probes at a fixed temperature [45,46].

- The dispersive component $\gamma_{l}^{d}$ of the surface energy of the solvent [44], the deformation polarizability $\alpha_{0}$ [47], and the topological index $\chi_{T}$ of the solvents [48,49].
- It was proven that these various methods cannot be considered as accurate quantitative methods that allow an accurate separation between the dispersive and polar free energies of adsorption and the only method theoretically well founded was that based on the deformation polarizability. However, this method was not well applied, because the authors did some approximations that led to wrong values of the polar contribution of the free energy of interaction between the solvents and the solid materials.

To better determine the two dispersive and polar free energies of solvents adsorbed on the PS*-b-*P4VP diblock copolymer, our new methodology [53,54,55,56,59,60] based on the London dispersion interaction energy was applied (Equation (12)):

(12) $$∆G_{a}^{d}\left(T\right)=-\frac{\alpha_{0S}\;}{\;H^{6}}\left[\frac{3N}{{2\left(4\pi \varepsilon_{0}\right)}^{2}}\left(\frac{\varepsilon_{S}\;\varepsilon_{X}}{\left(\varepsilon_{S}+\varepsilon_{X}\right)}\alpha_{0X}\right)\right]$$

where $\varepsilon_{0}$ is the dielectric constant of vacuum, $\alpha_{0S}\;$and $\alpha_{0X}$ are the respective deformation polarizabilities of the solid material denoted by S and the organic solvent denoted by X, and $\varepsilon_{S}$ and $\varepsilon_{X}$ are their corresponding ionization energies. Meanwhile, H designates the separation distance between the organic solvents and the copolymer.

By combining Equations (6), (8), and (12), the author obtained the following relation:

(13) $$RTlnVn=\frac{\alpha_{0S}\;}{\;H^{6}}\left[\frac{3N}{{2\left(4\pi \varepsilon_{0}\right)}^{2}}\left(\frac{\varepsilon_{S}\;\varepsilon_{X}}{\left(\varepsilon_{S}+\varepsilon_{X}\right)}\alpha_{0X}\right)\right]-∆G_{a}^{p}\left(T\right)+C\left(T\right)$$

A chromatographic interaction parameter $P_{SX}$ was chosen:

(14) $$P_{SX}=\frac{\varepsilon_{S}\;\varepsilon_{X}}{\left(\varepsilon_{S}+\varepsilon_{X}\right)}\alpha_{0X}$$

In the case of non-polar molecules such as n-alkanes, the variations in RTlnVn of the solvents were obtained from Equation (15):

(15) $$\left\{\begin{array}{c} RTlnVn\left(non-polar\right)=A\left[\frac{3N}{{2\left(4\pi \varepsilon_{0}\right)}^{2}}P_{SX}\left(non-polar\right)\right]+C(T)\\ A=\frac{\alpha_{0S}\;}{\;H^{6}}\;\;\;\;\;\;\;\;\;\;\;\;\;\;\;\;\;\;\;\;\;\;\;\;\; \end{array}\right.$$

where A is the slope of the non-polar straight line, which is a function of the separation distance between the solid surface and organic molecules. 

The free polar energy $∆G_{a}^{p}\left(polar\right)$ of polar molecules adsorbed on the diblock copolymer were then obtained as a function of the temperature using Equation (16):

(16) $$∆G_{a}^{p}\left(T,\;polar\right)=RTlnVn\;\left(T,\;polar\right)-A\left[\frac{3N}{{2\left(4\pi \varepsilon_{0}\right)}^{2}}P_{SX}\left(polar\right)\right]-C(T)$$

#### 3.3.4. Lewis’s Acid–Base Parameters of PS*-b-*P4VP Diblock Copolymer 

The experimental determination of $∆G_{a}^{p}\left(T,\;polar\right)$ was used to obtain the values of polar enthalpy $\left(-∆H_{a}^{p}\right)$ and entropy $\left(-∆S_{a}^{p}\right)$ of polar probes adsorbed on the PS-*b*-P4VP diblock copolymer. Relation 17 is used when the linearity of $∆G_{a}^{p}\left(T\right)$ is satisfied.

(17) $$∆G_{a}^{p}\left(T\right)=∆H_{a}^{p}-T∆S_{a}^{p}$$

However, in many cases of the interaction between polymers and organic solvents, the linearity was not insured. The values $\left(-∆H_{a}^{p}(T)\right)$ $\left(-∆S_{a}^{p}(T)\right)$ were then determined from the following thermodynamic equations:

(18) $$\left\{\begin{array}{c} ∆H_{a}^{sp}\left(T\right)=\frac{\partial \left(\frac{∆G_{a}^{sp}\left(T\right)}{T}\right)}{\partial \left(\frac{1}{T}\right)}\;\;\;\;\;\;\;\;\;\;\;\\ ∆S_{a}^{sp}(T)=-\left(\frac{\partial \left(∆G_{a}^{sp}\left(T\right)\right)}{\partial T}\right) \end{array}\right.$$

The classic relations, relation 19, were used to determine the variations in the Lewis acid–base parameters of the PS-*b*-P4VP copolymer as a function of the temperature. Their enthalpic (*K_A_*, *K_D_)* and entropic ($\omega_{A}$, $\omega_{D}$) acid–base parameters were therefore determined:

(19) $$\left\{\begin{array}{c} \left(-∆H^{p}\right)=\;K_{A}\times DN^{\prime }+K_{D}\times AN^{\prime }\;\;\\ \left(-∆S_{a}^{p}\right)=\omega_{A}\times {DN}^{\prime }+\omega_{D}\times {AN}^{\prime } \end{array}\right.$$

where $DN^{\prime }$ and $AN^{\prime }$ are, respectively, the corrected electron donor and acceptor numbers of the polar molecule [96,97].

#### 3.3.5. Lewis’s Acid–Base Surface Energies of PS*-b-*P4VP Copolymer

The application of the Hamieh thermal model led to the London dispersive surface energy $\gamma_{s}^{d}(T)$ of the PS-*b*-P4VP copolymer against the temperature. However, the total surface energy $\gamma_{s}^{tot.}$ of a solid surface is given by

(20) $$\gamma_{s}^{tot.}=\gamma_{s}^{d}+\gamma_{s}^{p}$$

where $\gamma_{s}^{p}=\gamma_{s}^{AB}$ represents the polar (or acid–base) contribution of the surface energy.

The determination of $\gamma_{s}^{p}$ was obtained using Van Oss et al.’s method [98]; it was possible to determine $\gamma_{s}^{p}$ of the PS-*b*-P4VP copolymer. The polar surface energy of the copolymer is given by

(21) $$\gamma_{s}^{p}=2\sqrt{\gamma_{s}^{+}\gamma_{s}^{-}}$$

where $\gamma_{s}^{+}$ and $\gamma_{s}^{-}$ are, respectively, the Lewis acid and base surface energies of the solid material.

Van Oss et al. [98] used two solvents such as ethyl acetate (base *B*) and dichloromethane (acid *A*), characterized by the following parameters:

(22) $$\left\{\begin{array}{c} \gamma_{A}^{+}=5.2\;\mathrm{mJ}/m^{2},\;\gamma_{A}^{-}=0\;\;\;\;\;\\ \gamma_{B}^{+}=0,\;\gamma_{B}^{-}=19.2\;\mathrm{mJ}/m^{2} \end{array}\right.$$

Knowing that the polar free energy $∆G_{a}^{p}\left(T\right)$ of the polar solvents are given by

(23) $$∆G_{a}^{p}\left(T\right)=2Na(T)\left(\sqrt{\gamma_{l}^{-}\gamma_{s}^{+}}+\sqrt{\gamma_{l}^{+}\gamma_{s}^{-}}\right)$$

the Lewis acid and base surface energies of the copolymer can therefore be obtained from Equation (24):

(24) $$\left\{\begin{array}{c} \begin{array}{c} \gamma_{s}^{+}\left(T\right)\;=\frac{{\left[∆G_{a}^{p}\left(T\right)\left(B\right)\right]}^{2}}{{4N}^{2}{\left[a_{B}(T)\right]}^{2}\gamma_{B}^{-}}\; \end{array}\;\\ \gamma_{s}^{-}\left(T\right)=\frac{{\left[∆G_{a}^{p}\left(T\right)\left(A\right)\right]}^{2}}{{4N}^{2}{\left[a_{A}(T)\right]}^{2}\gamma_{A}^{+}}\; \end{array}\right.$$

Equations (20), (21), and (24) led to the values of the polar and total surface energies of the copolymer.

## 4. Conclusions

The surface thermodynamic properties of the PS-*b*-P4VP diblock copolymer were determined as a function of the temperature using the inverse gas chromatography technique at infinite dilution and applying the new methodology based on the Hamieh thermal model, consisting in the accurate determination of the London dispersive surface energy of the copolymer, and on the London dispersion interaction equation, allowing the separation between the dispersive and polar free energies of the adsorption of organic solvents on the PS-*b*-P4VP diblock copolymer. An important effect of the temperature on the Lewis acid–base properties of the copolymer was shown. Two glass transition temperatures of the copolymer were highlighted, the first one at 105 °C corresponding to the glass transition of PS polymer blocks and the second one at 148 °C being relative to that of P4VP blocks.

The variations in the free energy of adsorption, the London dispersive surface energy, and the Lewis acid and base surface energies of the copolymer as a function of the temperature all showed the presence of the two glass transition temperatures.

It was also shown that the variations in the different Lewis acid–base parameters were non-linear with the presence of two maxima at the transition temperatures. An important basic character of the copolymer was observed and varying with the temperature. The calculations of the average values of the Lewis acid–base parameters showed a copolymer surface 7.5 times more basic than acidic.

The separation distance between the organic molecules and the PS-*b*-P4VP diblock copolymer was determined and non-linear variations were also noticed with two minima observed at the glass transitions. An average value of the separation distance equal to 5.5Å was obtained. Our new methodology allowed for obtaining the variations in the acid $\gamma_{s}^{+}$ and base $\gamma_{s}^{-}$ surface energies, the polar acid–base surface energy $\gamma_{s}^{p}$, the London dispersive surface energy $\gamma_{s}^{d}$, and the Lifshitz—van der Waals surface energy $\gamma_{s}^{LW}$ (mJ/m^2^) of the PS-*b*-P4VP diblock copolymer as well as the polar surface energy of the solvents and their work of adhesion as a function of temperature. 

## Figures and Tables

**Table 1:** Equations of $\gamma_{s}^{d}(T)$ of the PS-*b*-P4VP diblock copolymer with the linear regression coefficients in the corresponding temperature interval.

$\mathrm{Equation}\;\mathrm{of}\;\gamma_{s}^{d}(T)$	R^2^	Temperature Interval
$\gamma_{s}^{d}\left(T\right)$ = 7.10^−4^*T^2^* − 0.324*T* + 66.3	0.9983	313.15K–378.15K
$\gamma_{s}^{d}\left(T\right)$ = 1.7.10^−2^*T^2^* − 13.733*T* + 2806	0.9910	378.15K–421.15K
$\gamma_{s}^{d}\left(T\right)$ = 9.1.10^−3^*T^2^* − 8.465*T* + 1979	0.9914	421.15K–473.15K

**Table 2:** Equations of the average polar free energy ($-∆G_{a}^{p}\left(T\right))$ and average values of the polar entropy and enthalpy of adsorption with the corresponding linear regression coefficients.

Solvent	$\mathrm{Equation}\;\mathrm{of}\;(-∆G_{a}^{p}\left(T\right))$ (kJ/mol)	$\left(-∆S_{a}^{p}\right)$ (J/k.mol)	$\left(-∆H_{a}^{p}\right)$ (kJ/mol)	R^2^
CHCl_3_	($-∆G_{a}^{p}\left(T\right))$ = −0.0086*T* + 8.083	8.6	8.083	0.7739
CH_2_Cl_2_	($-∆G_{a}^{p}\left(T\right))$ = −0.0079*T* + 14.074	7.9	14.074	0.7529
THF	($-∆G_{a}^{p}\left(T\right))$ = −0.0186*T* + 1.733	18.6	1.733	0.8626
MeCN	($-∆G_{a}^{p}\left(T\right))$ = −0.018*T* + 24.538	18.0	24.538	0.9422
CCl_4_	($-∆G_{a}^{p}\left(T\right))$ = −0.0195*T* + 13.095	19.5	13.095	0.8499
Ethyl acetate	($-∆G_{a}^{p}\left(T\right))$ = −0.0071*T* + 8.773	7.1	8.773	0.423
Ethanol	($-∆G_{a}^{p}\left(T\right))$ = −0.020*T* + 18.952	20.0	18.952	0.8176
Acetone	($-∆G_{a}^{p}\left(T\right))$ = 0.0084*T* + 13.14	8.4	13.140	0.3215

**Table 3:** Average values of the various enthalpic and entropic Lewis acid–base parameters of the PS*-b-*P4VP diblock copolymer with the corresponding linear regression coefficients.

Lewis’s Acid–Base Parameter	Average Values	R^2^
$K_{A}$	0.092	0.8556
$K_{D}$	0.693	0.8556
$K_{D}$ */* $K_{A}$	7.533	0.8556
$\left(K_{D}+K_{A}\right)$	0.785	0.8556
$10^{3}\times \omega_{A}\;$	0.06	0.6732
$10^{3}\times \omega_{D}$	1.0	0.6732
$\omega_{D}/\omega_{A}$	18.18	0.6732
$10^{3}\times (\omega_{D}+\omega_{A})$	1.06	0.6732

**Table 4:** Values of the polar acid and base surface energies $\gamma_{s}^{+}$, $\gamma_{s}^{-}$, $\gamma_{s}^{p}$, $\gamma_{s}^{d}$, and $\gamma_{s}^{tot.}$ (mJ/m^2^) of the PS*-b-*P4VP diblock copolymer as a function of the temperature.

T(K)	$\gamma_{s}^{-}$	$\gamma_{s}^{+}$	$\gamma_{s}^{p}$	$\gamma_{s}^{d}$	$\gamma_{s}^{tot.}$
313.15	43.33	15.82	52.36	28.24	80.61
323.15	51.05	14.93	55.21	29.63	84.85
333.15	59.29	14.07	57.77	31.00	88.77
343.15	68.08	13.25	60.07	32.33	92.40
353.15	76.49	12.40	61.59	33.29	94.88
363.15	82.96	12.28	63.84	34.79	98.63
368.15	81.51	11.81	62.05	30.08	92.13
373.15	79.96	11.59	60.87	27.26	88.13
378.15	82.02	12.02	62.79	32.75	95.54
383.15	73.70	11.33	57.81	29.72	87.52
393.15	58.03	11.62	51.94	25.17	77.11
403.15	65.57	11.81	55.66	25.31	80.97
413.15	77.12	12.34	61.69	27.09	88.78
421.15	79.58	11.54	60.60	29.45	90.05
423.15	78.67	11.55	60.30	26.54	86.84
433.15	51.16	8.51	41.73	19.85	61.58
438.15	51.42	8.06	40.72	16.93	57.65
443.15	50.79	7.66	39.45	15.46	54.92
453.15	52.39	7.57	39.82	13.05	52.87
463.15	54.15	7.25	39.63	11.72	51.35
473.15	53.70	6.89	38.48	10.68	49.16

## Data Availability

Data are contained within the article and Supplementary Materials.

## Supplementary Materials

The following supporting information can be downloaded at: https://www.mdpi.com/article/10.3390/molecules29204812/s1, Table S1: Values of RTlnVn (kJ/mol) of n-alkanes adsorbed on the PS-P4VP copolymer as a function of temperature. Table S2: Values of RTlnVn (kJ/mol) of polar solvents adsorbed on the PS-P4VP copolymer as a function of temperature. Table S3: Values of $-∆G_{a}^{p}\left(T\right)$ (kJ/mol) of polar solvents adsorbed on the PS-P4VP copolymer as a function of temperature. Table S4: Values of $-∆H_{a}^{p}\left(T\right)$ (kJ/mol) of polar solvents adsorbed on the PS-P4VP copolymer as a function of temperature. Table S5: Values of $-∆S_{a}^{p}\left(T\right)$ (J.mol^−1^.K^−1^) of polar solvents adsorbed on the PS-P4VP copolymer as a function of temperature.
