# Supplementary material for: Exploring the Application of Advanced Chromatographic Methods to Characterize the Surface Physicochemical Properties and Transition Phenomena of Polystyrene-b-poly(4-vinylpyridine)
Source: Molecules. 2024 Oct 11;29(20):4812. doi: 10.3390/molecules29204812 (PMC11510071; doi:10.3390/molecules29204812)
Supplement: Supplementary file 1 [file molecules-29-04812-s001.zip › molecules-3238258-supplementary.pdf]

Supplementary Materials

Exploring the Application of Advanced Chromatographic Methods to Characterize the Surface Physicochemical Properties and Transition Phenomena of Polystyrene-*b*-Poly(4-vinylpyridine)

Tayssir Hamieh[1,2]

[1]Faculty of Science and Engineering, Maastricht University, P.O. Box 616, 6200 MD Maastricht, Netherlands
[2]Laboratory of Materials, Catalysis, Environment and Analytical Methods (MCEMA), Faculty of Sciences, Lebanese University, Hadath, Lebanon

Correspondence: Email: t.hamieh@maastrichtuniversity.nl, Tel.: +31 6 5723 9324.

Table S1. Values of $RTlnVn$ (kJ/mol) of n-alkanes adsorbed on PS-P4VP copolymer as a function of temperature.

T(K)	C6	C7	C8	C9
313.15	3.093	5.125	7.157	9.189
323.15	2.494	4.579	6.664	8.750
333.15	1.895	4.034	6.173	8.311
343.15	1.296	3.488	5.680	7.872
353.15	0.697	2.943	5.088	7.434
363.15	0.097	2.396	4.494	6.893
368.15	-0.103	2.250	4.200	6.621
373.15	-0.283	2.100	3.950	6.350
378.15	-0.332	1.952	3.867	6.293
383.15	0.113	2.054	4.232	6.475
393.15	0.923	2.742	4.877	6.929
403.15	0.352	2.111	4.156	6.522
413.15	-0.478	1.552	3.445	5.987
421.15	-0.906	1.263	3.102	5.542
423.15	-0.822	1.301	3.185	5.605
433.15	0.525	2.125	3.822	6.211
438.15	0.415	1.925	3.712	6.025
443.15	0.214	1.675	3.462	5.697
453.15	-0.268	1.146	2.952	5.087
463.15	-0.742	0.722	2.456	4.552
473.15	-1.123	0.345	2.022	4.015

Table S2. Values of $RTlnVn$ (kJ/mol) of polar solvents adsorbed on PS-P4VP copolymer as a function of temperature.

T(K)	CH_2Cl_2	$CHCl_3$	CCl_4	Acetone	MeCN	THF	Ethanol	Ethyl acetate
313.15	3.189	5.565	5.031	6.177	9.953	6.136	5.560	7.897
323.15	2.854	4.762	4.256	5.401	8.949	5.055	5.123	7.000
333.15	2.519	3.959	3.481	4.625	7.946	3.974	4.687	6.103
343.15	2.185	3.156	2.706	3.849	6.943	2.893	4.250	5.206
353.15	1.750	2.353	1.932	3.073	5.939	1.612	3.813	4.309
363.15	1.415	1.750	1.157	2.296	4.936	0.731	3.276	3.712
368.15	1.262	1.554	0.920	2.012	4.623	0.523	3.011	3.457
373.15	1.080	1.422	0.754	1.887	4.372	0.354	2.784	3.211
378.15	0.988	1.322	0.681	1.855	4.223	0.287	2.662	3.041
383.15	1.245	1.701	0.942	2.221	4.375	0.622	2.822	3.353
393.15	2.254	2.752	1.811	2.788	5.258	1.334	3.669	4.225
403.15	1.654	2.131	1.322	2.222	4.622	0.754	3.345	3.782
413.15	0.958	1.404	0.702	1.495	3.785	0.165	2.756	3.245
421.15	0.491	0.912	0.222	1.022	3.334	-0.123	2.254	2.722
423.15	0.522	0.988	0.322	1.103	3.407	-0.054	2.341	2.802
433.15	1.225	1.885	0.855	1.840	4.022	0.524	2.912	3.442
438.15	1.044	1.664	0.755	1.694	3.850	0.345	2.642	3.222
443.15	0.722	1.422	0.546	1.484	3.665	0.124	2.345	2.944
453.15	0.225	0.985	0.128	1.022	3.247	-0.212	1.922	2.422
463.15	-0.225	0.514	-0.228	0.610	2.778	-0.523	1.456	1.966
473.15	-0.723	0.154	-0.556	0.238	2.384	-0.756	1.055	1.556

Table S3. Values of $(-\Delta G_a^p(T)$ (kJ/mol) of polar solvents adsorbed on PS-P4VP copolymer as a function of temperature.

T(K)	CH_2Cl_2	$CHCl_3$	CCl_4	Acetone	MeCN	THF	Ethanol	Ethyl acetate
313.15	5.391	2.406	9.547	14.971	7.568	5.636	12.319	10.550
323.15	5.264	2.243	9.540	14.780	7.205	5.882	12.219	11.508
333.15	5.136	2.079	9.533	14.589	6.842	6.127	12.118	12.465
343.15	5.009	1.916	9.526	14.398	6.479	6.373	12.018	13.423
353.15	4.869	1.756	9.483	14.161	5.893	6.604	11.876	14.299
363.15	4.870	1.571	9.332	13.791	5.625	6.673	11.909	14.965
368.15	4.787	1.494	9.091	13.490	5.498	6.516	11.678	14.870
373.15	4.753	1.473	8.997	13.239	5.397	6.382	11.443	14.764
378.15	4.853	1.594	9.173	13.302	5.534	6.461	11.484	14.990
383.15	4.725	1.402	8.953	12.831	5.326	6.107	11.186	14.244
393.15	4.807	1.434	8.362	12.468	4.983	5.973	10.843	12.701
403.15	4.870	1.587	8.539	12.603	5.114	6.337	11.162	13.566
413.15	5.001	1.744	8.789	12.798	5.437	6.614	11.637	14.783
421.15	4.855	1.621	8.644	12.667	5.487	6.457	11.436	15.075

423.15	4.863	1.654	8.656	12.670	5.487	6.476	11.447	15.003
433.15	4.193	0.913	7.403	11.098	4.307	5.450	9.971	12.156
438.15	4.091	0.946	7.357	11.017	4.238	5.298	9.845	12.216
443.15	3.998	0.930	7.231	10.886	4.136	5.145	9.632	12.170
453.15	3.992	0.985	7.139	10.811	4.204	5.149	9.463	12.418
463.15	3.926	1.066	7.087	10.680	4.278	5.085	9.353	12.685
473.15	3.846	1.080	6.904	10.433	4.283	4.957	9.105	12.691

Table S4. Values of $(-\Delta H_a^p(T)$ (kJ/mol) of polar solvents adsorbed on PS-P4VP copolymer as a function of temperature.

T(K)	CH_2Cl_2	$CHCl_3$	CCl_4	Acetone	MeCN	THF	Ethanol	Ethyl acetate
313.15	9.461	7.614	5.961	13.401	18.937	-1.960	15.732	-17.474
323.15	9.461	7.614	7.870	15.310	18.937	-1.960	15.732	-17.474
333.15	9.461	7.614	9.839	17.279	18.937	-1.960	15.732	-17.474
343.15	9.461	7.614	11.868	19.308	18.937	-1.960	15.732	-17.474
353.15	9.461	7.614	13.956	21.396	14.735	-1.960	15.732	-17.474
363.15	18.930	7.614	16.105	23.545	14.735	19.383	40.960	-17.474
368.15	12.348	7.614	17.202	24.642	14.735	10.607	31.087	2.876
373.15	5.676	-12.902	18.314	25.754	14.735	1.712	21.080	22.891
378.15	-1.085	10.764	35.235	48.557	25.244	-7.304	10.937	43.176
383.15	5.864	34.745	28.003	37.519	19.154	19.580	34.742	63.731
393.15	5.087	-4.649	13.253	15.006	6.733	0.173	19.216	76.000
403.15	4.291	-4.649	-1.877	-8.087	-6.008	-19.735	3.290	-27.300
413.15	3.475	-4.649	-17.387	-9.496	-19.069	4.412	-13.036	-13.694
421.15	29.936	28.606	-30.068	33.888	65.841	30.443	86.548	55.720
423.15	27.572	28.606	55.281	44.863	60.100	37.028	80.469	73.282
433.15	15.584	28.606	55.281	63.452	30.986	24.636	49.642	94.520
438.15	9.484	-1.088	55.281	15.853	16.174	14.617	33.958	14.817
443.15	3.315	-1.088	3.148	16.734	1.191	4.482	18.095	4.682
453.15	7.297	-1.088	4.941	18.527	-8.958	2.503	13.611	-15.933
463.15	7.297	-1.088	6.774	20.360	-6.209	5.252	20.026	12.407
473.15	7.297	-1.088	8.646	22.232	-3.400	8.061	26.580	12.407

Table S5. Values of $(-\Delta S_a^p(T)$ (J.mol^{-1}.K^{-1}) of polar solvents adsorbed on PS-P4VP copolymer as a function of temperature.

T(K)	CH_2Cl_2	$CHCl_3$	CCl_4	Acetone	MeCN	THF	Ethanol	Ethyl acetate
313.15	13.0	16.6	-12.4	-16.8	36.3	-24.3	10.9	-89.7
323.15	13.0	16.6	-6.4	-10.8	36.3	-24.3	10.9	-89.7
333.15	13.0	16.6	-0.4	-4.8	36.3	-24.3	10.9	-89.7
343.15	13.0	16.6	5.6	1.2	36.3	-24.3	10.9	-89.7
353.15	13.0	16.6	11.6	7.2	25.1	-24.3	10.9	-89.7

363.15	48.4	16.6	17.6	13.2	25.1	19.2	88.8	-89.7
368.15	30.4	16.6	20.6	16.2	25.1	-4.8	61.8	-16.3
373.15	12.4	-21.6	23.6	19.2	25.1	-28.8	34.8	37.7
378.15	-5.6	41.4	60.7	87.4	45.9	-52.8	7.8	91.7
383.15	15.2	80.0	41.7	58.4	29.9	32.0	78.5	112.2
393.15	14.2	15.5	3.7	0.4	-2.1	-18.0	38.5	130.3
403.15	13.2	15.5	-34.3	-57.6	-34.1	-68.0	-1.5	-121.5
413.15	12.2	15.5	-51.2	-61.8	-66.1	9.1	-41.5	-55.3
421.15	170.2	63.9	-102.7	42.3	98.6	51.0	124.0	91.4
423.15	162.0	63.9	110.5	68.2	84.6	62.0	106.7	133.0
433.15	143.0	63.9	110.5	133.5	63.4	28.5	84.0	155.0
438.15	122.3	4.6	110.5	13.8	29.4	5.5	66.0	-8.7
443.15	114.0	4.6	-21.8	15.8	-4.6	-17.5	30.0	-31.7
453.15	97.2	4.6	-17.8	19.8	-47.3	-14.8	14.2	-77.7
463.15	7.3	4.6	-13.8	23.8	-41.3	-8.8	28.2	0.6
473.15	7.3	4.6	-9.8	27.8	-35.3	-2.8	42.2	0.6
